# Effect of In-Incorporation and Annealing on Cu_x_Se Thin Films

**DOI:** 10.3390/ma14143810

**Published:** 2021-07-08

**Authors:** Algimantas Ivanauskas, Remigijus Ivanauskas, Ingrida Ancutiene

**Affiliations:** Department of Physical and Inorganic Chemistry, Faculty of Chemical Technology, Kaunas University of Technology, 44249 Kaunas, Lithuania; algimantas.ivanauskas.88@gmail.com (A.I.); ingrida.ancutiene@ktu.lt (I.A.)

**Keywords:** SILAR, selenium, copper selenide, indium selenide

## Abstract

A study of indium-incorporated copper selenide thin-film deposition on a glass substrate using the successive ionic adsorption and reaction method (SILAR) and the resulting properties is presented. The films were formed using these steps: selenization in the solution of diseleniumtetrathionate acid, treatment with copper(II/I) ions, incorporation of indium(III), and annealing in an inert nitrogen atmosphere. The elemental and phasal composition, as well as the morphological and optical properties of obtained films were determined. X-ray diffraction data showed a mixture of various compounds: Se, Cu_0.87_Se, In_2_Se_3_, and CuInSe_2_. The obtained films had a dendritic structure, agglomerated and not well-defined grains, and a film thickness of ~90 μm. The band gap values of copper selenide were 1.28–1.30 eV and increased after indium-incorporation and annealing. The optical properties of the formed films correspond to the optical properties of copper selenide and indium selenide semiconductors.

## 1. Introduction

While the majority of the world’s energy supply is generated from non-renewable fossil fuels, such as oil, coal, and natural gas, there are a number of disadvantages in using them, such as rising prices, increasing environmental concerns over climate change, and security concerns due to dependency on imports from a limited number of countries that have substantial fossil fuel supplies. As a result, governments and businesses increasingly support renewable energies such as wind, hydroelectric, geothermal, biomass, and solar power.

Solar power is proven to be one of the most promising and popular renewable energy sources today since it has several advantages over other renewable energy sources. Solar power generates energy with very limited environmental impact. Its peak energy output matches the peak energy demand time, making it a perfect supplement for already existing power grids. Solar power plants are very easy to scale up, as the energy generating capacity is directly dependent on the plant’s size and the number of modules installed. Solar power production facilities can be installed in environmentally sensitive and remote locations, such as national parks and remote homes, where no power grid and other energy sources are available. Solar cells have no moving parts, thus they require little service and operate noise free. The worldwide production of solar power has been increasing exponentially during the last decades [1].

Copper indium selenide is a widely used thin-film substance in solar energy and photovoltaic applications. Over the years, it has attracted a lot of attention due to its desired physical properties. CuInSe_2_ has a low direct band gap (1.04 eV) [2], a high absorption coefficient (>10^5^ cm^−1^) [2], and a high thermal resilience [3]. It shows no performance degradation under intensive solar radiation [4]. Currently, maximum CuInSe_2_ efficiencies of 23.3% were achieved in thin-film layers, and 22.9% in solar cells [5]. Due to continuous improvements in the efficiency of CuInSe_2_ cells, it is now widely used in photovoltaic technologies.

CuInSe_2_ films can be formed using various deposition methods. Chemical deposition methods include spin-coating [6], electrochemical deposition [7], and chemical bath deposition [8], while physical deposition methods include electron beam evaporation [9], sputtering [10], molecular beam epitaxy [11], physical vapor deposition [12], printing [13], etc. Films which are formed using physical deposition methods are usually more uniform and of better quality; however, expensive, high-temperature, and low-pressure equipment is often needed. Furthermore, these methods offer low scalability for large-area coating and often require a toxic H_2_Se atmosphere to anneal. This results in a toxic work environment and lower cost efficiency due to wasted reagents. Chemical deposition methods are a more convenient way to deposit CuInSe_2_, due to not needing hazardous selenization processes and not requiring expensive instrumentation. In addition, it is much easier to make films with a large surface area. Thus, two wet chemical deposition methods, such as chemical bath deposition (CBD) [14,15] and successive ionic layer adsorption and reaction (SILAR) [16,17], are widely studied and used to deposit thin semiconductor films on various substrates. However, one of the drawbacks of the CBD method is the waste of the solution after each deposition. This also leads to the formation of a precipitate in the solution and complications in controlling the process. By using the SILAR method, thin-film deposition occurs by contacting the substrate with a chemical bath containing the appropriate ions, thus avoiding the formation of precipitate. D. Kishore Kumar et al. have shown [18] that the SILAR method provides phase purity of a tin selenide layer formed and used in solar cells. In addition, this method allows the thickness, morphology, and composition to be controlled, which is very important for the optical properties of a thin film [19].

The films formed using chemical deposition methods often require annealing to obtain a crystalline layer and improve properties.

The incorporation of elements has been found to be an effective way to modify the properties of semiconductors and improve the conversion efficiency of solar energy. Indium is considered to be one of the most efficient elements that can be used to improve the conductivity of thin films.

This work studies the deposition of In-incorporated Cu_x_Se films on a glass substrate using the three-step SILAR method and subsequent annealing in a nitrogen atmosphere. To understand the reaction pathways, composition, and properties of obtained films, X-ray diffraction (XRD), X-ray photoelectron spectroscopy (XPS), scanning electron microscopy (SEM), energy dispersive X-ray spectroscopy (EDX), atomic absorption spectroscopy (AAS), and ultraviolet-visible spectroscopy (UV-Vis) measurements were carried out.

## 2. Materials and Methods

### 2.1. Materials

All chemicals used are pure commercial reagents from Sigma–Aldrich (Sigma-Adrich Chemie GmbH, Taufkirchen, Germany): KHSO_3_ (≥98.0%), H_2_SeO_3_ (99.99% trace metals basis), CuSO_4_·5H_2_O (99.99% crystals and lumps), hydroquinone (≥99% flakes), and InCl_3_ (98% reagent grade).

Thomas^®^ environmental glass slides with one side sandblasted (20 × 20 mm^2^) were used for film deposition. 

### 2.2. Treatment Methods

Before the thin-film deposition process, glass slides were thoroughly cleaned with liquid soap, washed with distilled water, and then bathed ultrasonically in acetone using the Sonoswiss SW 3 H cleaner in sweep mode at 40 °C for 10 min. After cleaning, glass slides were dried before use.

In-incorporated Cu_x_Se films were deposited in three stages and then annealed. The first step was selenization at 60 °C for 180 min in diseleniumtetrathionate acid solution, which is produced by mixing of 1 mol/L KHSO_3_ and 0.4 mol/L H_2_SeO_3_ (1:1) [20]:

2H_2_SeO_3_ + 5KHSO_3_ → H_2_Se_2_S_2_O_6_ + 2K_2_SO_4_ + KHSO_4_ + 3H_2_O (1)

Elemental selenium is deposited by submerging glass substrate into H_2_Se_2_S_2_O_6_ solution, which decomposes into elemental selenium and seleniumtrithionate via reaction (2) [21]:

Se_2_S_2_O_6_^2−^ → Se + SeS_2_O_6_^2−^ (2)

The deposited selenium film was washed in distilled water, and then submerged in copper(II/I) ion solution at 40 °C for 10 or 20 min, and 60 °C for 5 or 10 min. Cu(II/I) salt solution was prepared using a solution of 0.4 mol/L CuSO_4_ and a reducing agent, hydroquinone (1%), therefore being a mixture of univalent and divalent copper salts. Cu(I) ions react with elemental selenium forming copper selenide via reaction (3):

Se + 2xCu^+^ → Cu_x_Se + xCu^2+^ (3)

**Figure 1:**
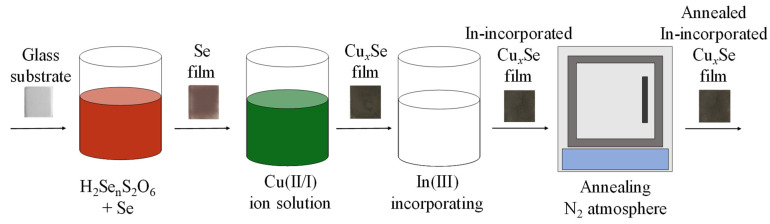
Schematic representation of In-incorporated Cu_x_Se film on glass synthesis.

Copper selenide films were washed in distilled water and then treated with 0.1 mol/L InCl_3_ solution at 40 °C for 20 min. Obtained films were washed again in distilled water and dried over CaCl_2_. The final step was film annealing at 100 °C for 12 h in an inert N_2_ atmosphere. The deposition scheme is shown in Figure 1.

### 2.3. Investigative Methods

X-ray diffraction analysis was conducted with a D8 Advance diffractometer (Bruker AXS, Karlsruhe, Germany) at 40 mA tube current and 40 kV voltage. 6° 1/min scanning speed was used with coupled two theta/theta scan type. Data was recorded with provided software package DIFFRAC.SUITE (Diffract.EVA.V4.3., Bruker, Karlsruhe, Germany) and analyzed using Search Match and Microsoft Office Excel.

X-ray photoelectron spectroscopy analysis was made using the upgraded Vacuum Generator (VG) ESCALAB MKII spectrometer (Waltham, MA, USA), with an added XR4 twin anode. Thermo VG Scientific Avantage software (5.918, Thermo Fisher Scientific, Waltham, MA, USA) was used to record data. Data were analyzed in Microsoft Office Excel.

Scanning electron microscopy analysis of In-incorporated Cu_x_Se film morphology was carried out with a Quanta 200 FEG microscope. Bruker XFlash 4030 detector (Bruker Corporation, Billerica, MA, USA) was used to perform energy dispersive X-ray analysis. Detected elements were quantified using ZAF method.

Atomic absorption spectroscopy analysis by atomic absorption spectrometer Shimadzu AA-7000 (Tokyo, Japan) was used to determine Se, Cu, and In amounts in In-incorporated Cu_x_Se films deposited on glass. A mixture of concentrated HNO_3_ and distilled water (1:1) was used to dissolve films. Acetylene–air combination was used as flame fuel. Amount (μmol/cm^2^) of Se, Cu, In in film was calculated using measured data.

To measure optical absorption spectra of 400–900 nm, Perkin Elmer Lambda 35 UV/VIS (Waltham, MA, USA) spectrometer with fitted diffuse reflectance sphere Labsphere RSA-PE-20 was used. Band gap E_g_ was calculated by plotting (αhν)^2^ against photon energy hν, extrapolating linear part of the plot until abscissa axis intersection ((αhν)^2^ = 0). Then, band gap value E_g_ was equal to hν at intersected part.

## 3. Results and Discussion

XRD, XPS, SEM/EDX, and AAS analyses were used in order to better understand the reaction pathways when using the SILAR deposition method, and to learn more about film composition during various formation steps.

### 3.1. XRD Analysis

**Figure 2:**
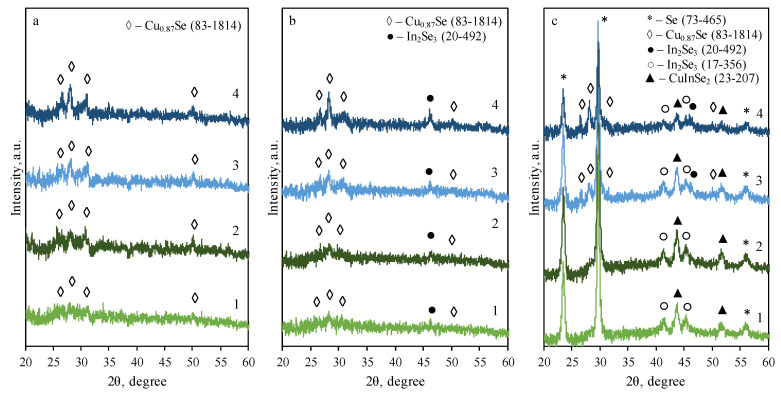
XRD patterns of Cu_x_Se and In-incorporated Cu_x_Se films on glass substrate: (**a**) treated with copper(II/I) ion solution, (**b**) treated with indium(III) salt solution, (**c**) annealed in nitrogen atmosphere. Treatment with copper(II/I) ion solution: 1—10 min at 40 °C; 2—20 min at 40 °C; 3—5 min at 60 °C; and 4—10 min at 60 °C.

Copper selenide and indium-incorporated Cu_x_Se films were formed on a glass substrate with a single-side matte finish. Figure 2 shows the XRD patterns of selenide films on glass for various formation stages. Graph (a) shows XRD patterns after the treatment of selenium films in copper(II/I) ion solution. Here, four peaks of hexagonal klockmannite Cu_0.87_Se (◊) at 2θ = 26.6, 28.1, 31.2, 50.0° (JCPDS: 83-1814) could be seen. Samples that were treated both longer (20 min vs. 10 min) and at higher temperature (60 °C vs. 40 °C) have slightly more intense peak values, indicating that more copper selenide was formed. This suggests that copper(I) ions react with elemental selenium, forming copper selenide as shown in Equation (3). Second graph (b) shows the XRD patterns after incorporation of indium(III). It could be seen from the graph that a brand-new peak of cubic indium selenide In_2_Se_3_ at 2θ = 46.0° (JCPDS: 20-492) appears in all XRD patterns. Indium(III) ions may react with copper selenide, forming indium selenide, according to reaction (4):

3CuSe + 2In^3+^ → In_2_Se_3_ + 3Cu^2+^ (4)

Ion exchange takes place due to the lower solubility of In_2_Se_3_ (solubility product is 5.6 × 10^−92^) than of CuSe (1.4 × 10^−36^) [22]. Similarly, samples that were treated longer and with higher temperatures have slightly more intense peaks, indicating that more indium selenide was formed.

Finally, the samples were annealed in an inert nitrogen atmosphere at 100 °C to obtain better crystalline properties. Annealing promotes reactions between solid phases and crystallization of amorphous phases:

2CuSe + In_2_Se_3_ → 2CuInSe_2_ + Se (5)

A number of new peaks can be seen in graph (c). Two intensive diffraction peaks at 2θ = 23.5, 29.7° and one weaker peak at 56.3° of hexagonal selenium (JCPDS: 73-465) are clearly observed. Evidently, amorphous elemental selenium crystallized during the annealing process. Two new peaks at 2θ = 41.2, 45.3° (JCPDS: 17-356) of the new phase of In_2_Se_3_ indium selenide appear. The new phase may change the previous phase of cubic indium selenide due to annealing. This is suggested by the disappeared peaks of cubic indium selenide in samples 1 and 2 and the smaller peaks in 3 and 4, compared to graph (b). The other two new peaks of CuInSe_2_ cubic copper indium selenide phase at 2θ = 43.7, 51.8° (JCPDS: 23-207) are found in the XRD patterns. A new phase of copper indium selenide may be formed during the solid phase reaction according to Equation (5). After annealing, the peaks of the hexagonal klockmannite phase are no longer found in samples 1 and 2. This could also be explained by Equation (5), as klockmannite was used in copper indium selenide formation.

### 3.2. XPS Analysis

**Figure 3:**
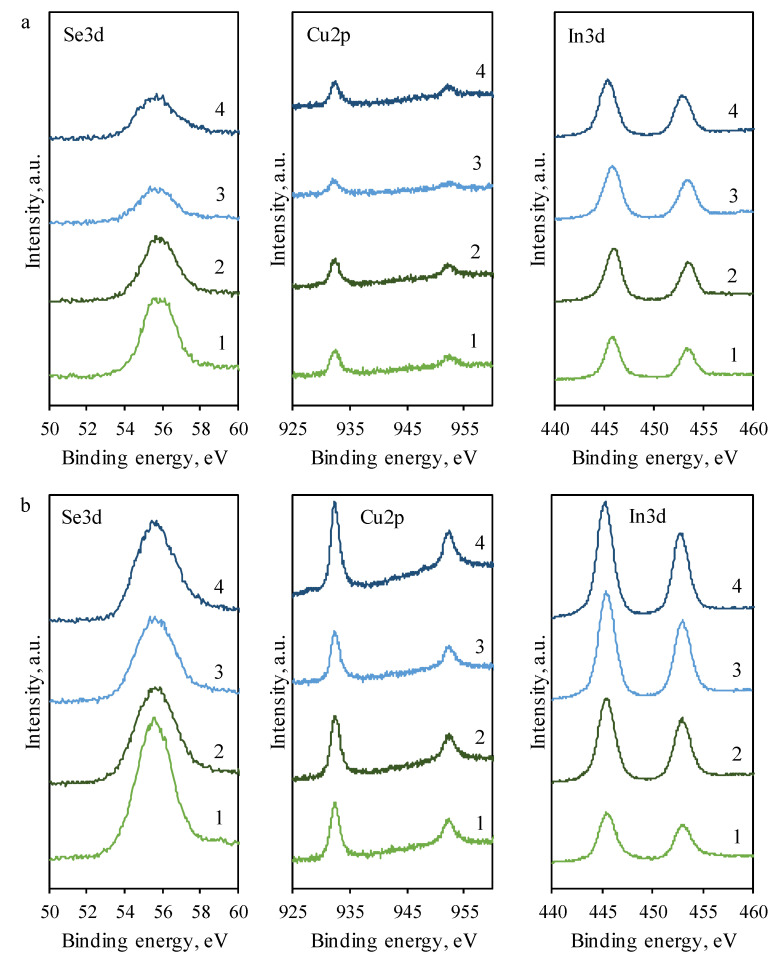
XPS spectra of In-incorporated Cu_x_Se non-etched (**a**) and etched (**b**) films. Treatment with copper(II/I) ion solution: 1—10 min at 40 °C; 2—20 min at 40 °C; 3—5 min at 60 °C; and 4—10 min at 60 °C.

The high-resolution XPS spectra are shown in Figure 3. Spectra were recorded at three regions corresponding to Se, Cu, and In. The data are presented in graph (a) for non-etched samples and in graph (b) for 30 s etched samples using an argon gas gun. Etching was necessary because the formed oxides, salt residues from solutions, and other residues could have been on the film surface.

The peaks corresponding to the Se3d_3/2_ spectra region have binding energies of 55.5–55.8 eV. These peaks correspond to elemental-selenium-binding energy values of 55.7 eV [23]. The peaks in Cu2p_1/2_ and Cu2p_3/2_ regions are slightly more intense in samples treated with a solution of copper(II/I) ions at a higher temperature, demonstrating a greater amount of deposited copper-containing compounds. This can be seen better on etched samples. The high-resolution spectra indicate that the binding energy values are 932.2–932.4 eV for the Cu2p_3/2_ region, which equates to CuSe (932.27 eV) [24], CuInSe_2_ (931.8–932.49 eV) [25], Cu_2_Se (931.9–932.5 eV) [26], and Cu_2_O (932.3–932.5 eV) [27,28]. The peaks at 952.2–952.4 eV found in the Cu2p_1/2_ region correspond to CuInSe_2_ (952.31 eV) [25] and Cu_2_O (952.5 eV) [28]. The In3d_5/2_ spectra region has the peaks at 445.1–445.9 eV, and these binding energy values correspond to In_2_Se_3_ (445.1 eV) [29], In(OH)_3_ (445.0–445.2 eV) [30], and InCl_3_ (445.9 eV) [31]. The NIST XPS does not have any entries matching the In3d_3/2_ spectra region, making results inconclusive. All XPS spectra show that all the samples that were etched have more intense peaks than non-etched samples. This is especially noticeable in the peak values of Cu2p and, to a lesser degree, In3d. This suggests that the deeper films contain more Se, Cu, and In, while the surface of films contain more impurities, residues of salts, and oxides. The full spectrum of XPS analysis revealed some impurity elements, O and Cl. The presence of oxygen can be explained by exposure to the atmosphere and the formation of oxides, as well as hydrolysis. The residual element Cl may come from the InCl_3_ precursor solution.

In our opinion, Cu_2_Se could be formed on the surface due to a dense selenide film, which prevents the penetration of Cu(I) ions and interaction with elemental selenium. The formed Cu_x_Se distinguishes the reacting substances (Cu^+^ and Se) from one another, thus the subsequent formation process is only possible due to the diffusion of reactants through the selenide film. Consequently, Cu_2_Se could form on the surface of the film. In a deeper layer, the formation of CuSe is possible due to the solid-phase reaction of copper selenide with selenium. Elemental selenium as an oxidizer and Cu_x_Se react toward a decrease in the x value via reactions (6) and (7):

yCu_x_Se + (x − y)Se → xCu_y_Se (6)

Cu_2_Se + Se → 2CuSe (7)

### 3.3. SEM/EDX Analysis

The color of the prepared films on the glass substrate changes from transparent to red during reaction (2), when the elemental selenium is deposited, and to a dark gray when copper selenide and indium-incorporated copper selenide films are formed (reactions (3)–(5)).

**Figure 4:**
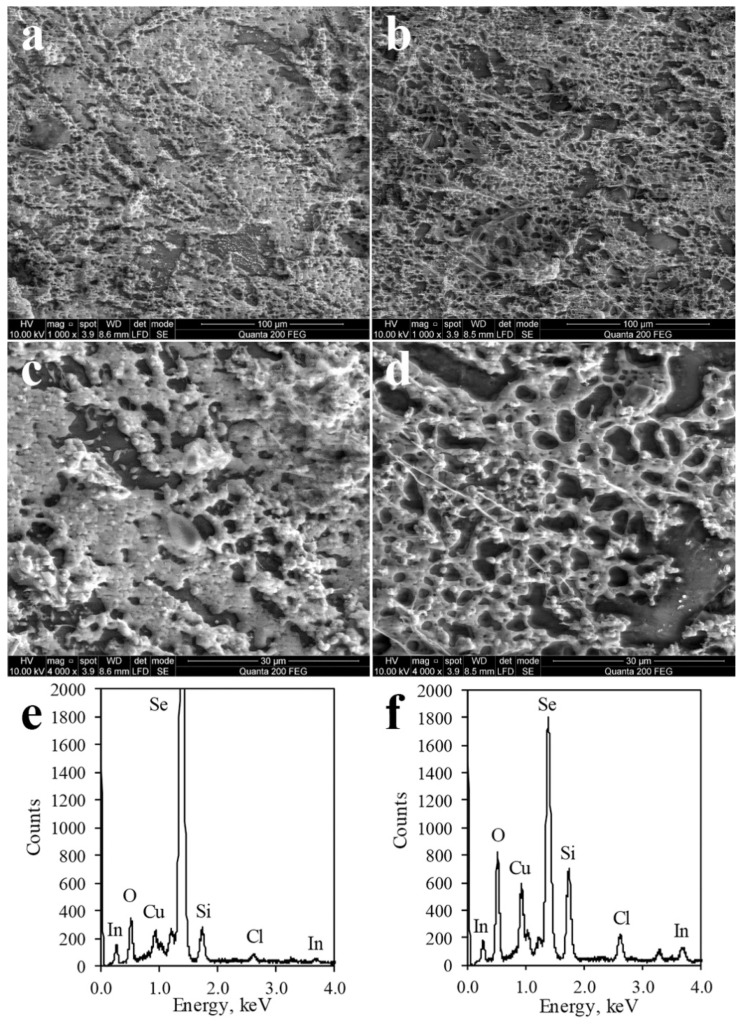
SEM images (**a**–**d**) and EDX spectra (**e**,**f**) of In-incorporated Cu_x_Se film on glass. SEM images are 4000× (**a**,**c**) and 1000× (**b**,**d**) magnifications. Treatment with copper(II/I) ion solution: (**a**,**c**,**e**) 10 min at 40 °C and (**b**,**d**,**f**) 10 min at 60 °C.

Structural features and surface morphology of the deposited films were analyzed using SEM, images from which are shown in Figure 4 graphs (a,b,c,d). Magnifications of 1000 (b,d) and 4000 (a,c) were used. The micrographs show a compact structure of films composed of single-type grains. The individual grains have a dendritic structure; grains are agglomerated and not well defined. 

EDX spectroscopy was used to study the elemental composition of obtained films. Peaks in EDX spectra shown in Figure 4 graphs (e,f) indicate atomic mass ratios of each element in the film. EDX spectra shows that the surface of the film, which was treated with copper(II/I) ion solution at a higher temperature (60 °C vs. 40 °C), contains more copper, a similar amount of indium, and less selenium. The lower peak of selenium could be explained by the formation of more copper containing compounds, such as Cu_0.87_Se and CuInSe_2_, due to a higher temperature and covering of elemental selenium. Besides selenium, copper, and indium, traces of other elements such as oxygen, chlorine, and silicon were found. The presence of oxygen may be explained by the dendritic film structure absorbing oxygen from the atmosphere. Silicon is part of the glass substrate itself, and the residual amount of chlorine may be brought in during the step of In-incorporation.

**Figure 5:**
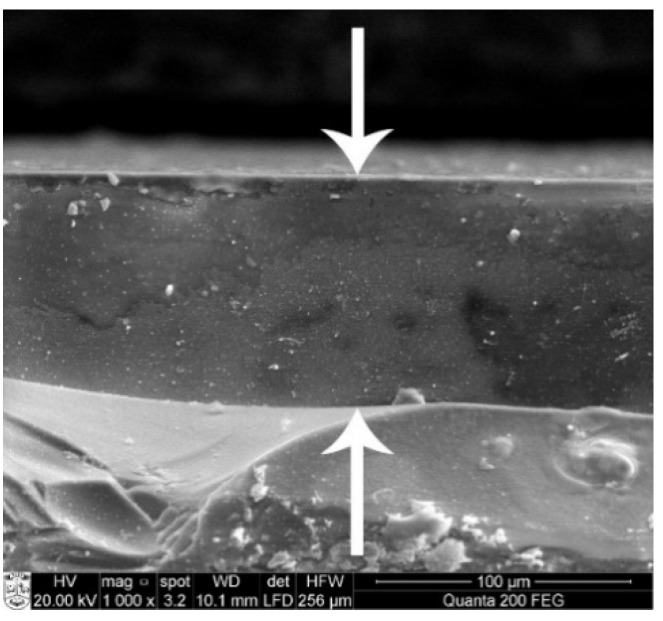
SEM cross-section image of In-incorporated Cu_x_Se annealed film on glass. Treatment with copper(II/I) ion solution was 10 min at 60 °C.

To estimate the thickness of In-incorporated copper selenide films, cross-section images were taken. The micrographs revealed the film size to be similar for all samples, about 90 μm (Figure 5). It is possible that the temperature and duration of the initial deposition of selenium film determines the thickness of the film.

### 3.4. AAS Analysis

The AAS analysis method was used to assess atomic amounts of Se, Cu, and In in obtained films. The data is shown in Table 1 and it coincides with the data obtained using the XRD, XPS, and EDX methods. All samples have similar amounts of selenium because all of the samples were deposited using the same selenization temperature and duration. The temperature and duration of the treatment with copper(II/I) ion solution have a major effect on the amounts of copper in the formed indium-incorporated copper selenide films. A longer treatment with copper ion solution and an increased temperature allows more copper to react with deposited elemental selenium, resulting in copper-rich films. The sample that was treated for 10 min at 40 °C had less copper than the 20 min one, additionally the sample treat for 5 min at 60 °C had less copper compared to the 10 min samples. Likewise, the 40 °C sample (0.50 μmol/cm^2^) had less copper than the 60 °C sample (0.78 μmol/cm^2^), both samples were treated for 10 min. The AAS analysis data lines up with the XRD data shown in Figure 2. A longer treatment with copper(II/I) ion solution at higher temperatures yields films with a larger amount of copper selenide (hexagonal klockmannite, Cu_0.87_Se), because the peaks are more intense. Furthermore, all samples have similar amounts of indium because of the same conditions of indium(III) incorporation.

### 3.5. Optical Properties

Optical transitions in semiconductors play an important role in their characterization. Optical properties were studied by measuring the absorption spectra of films and calculating the Tauc plot. It shows the energy of the light *hν* versus absorption quantity (*αhν*)*^n^*, where α is the absorption coefficient of the studied film material. In order to calculate the energy bandgap (*E_g_*) of the obtained films the Tauc plot was used [32,33,34]:

(8) $$\alpha =\left(\frac{A}{h\nu }\right){\left(h\nu -E_{g}\right)}^{n}$$

where *A* is the parameter that depends on transition chance and *n* is the value that depends on transition type. The number *n* = 2 denotes direct band gap transition and *n* = ½ denotes indirect band gap transition. The resulting plot has a distinct linear segment that indicates the beginning of the light absorption spectra. The intersection of the extrapolated linear part with the abscissa axis shows the band gap value *E_g_*. Extrapolating the linear region to the abscissa yields the energy of the optical band gap of the material. 

The band gap values of the prepared semiconductor films were found to be in a range of 1.28–1.48 eV. This corresponds to the absorption start at 840–970 nm. These band gap values are similar to the compounds identified by XPS and XRD analysis. The band gap values of various semiconductors are shown in Table 2.

**Figure 6:**
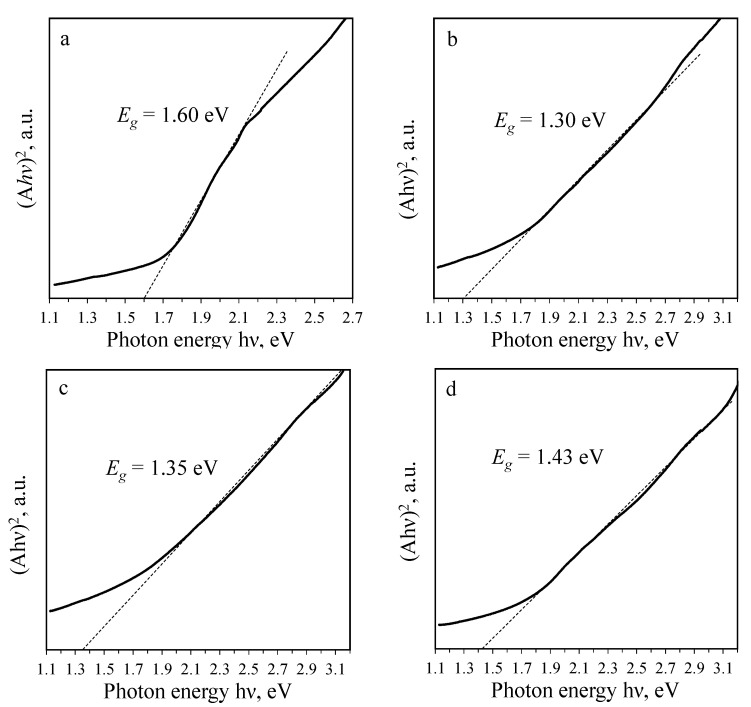
Tauc plot of obtained films on glass: (**a**) after selenization, (**b**) after treatment with copper(II/I) ion solution at 60 °C for 5 min, (**c**) after In-incorporation, and (**d**) after annealing.

Firstly, the glass substrate was submerged into a selenopolythionic acid solution and a reddish elemental selenium film was formed. The band gap of selenium film was 1.60 eV (Figure 6a) and corresponded to the values found in the literature. The band gap values of formed copper selenide films were in the range of 1.28–1.30 eV (Figure 6b) and are similar to the Cu_2_Se band gap *E_g_* = 1.1–1.27 eV and Cu_2−x_Se band gap *E_g_* = 1.4–2.2 eV (Table 2). After incorporation of In(III), the band gap values of those films increased to *E_g_* = 1.32–1.38 eV (Figure 6c). After annealing, the band gap values of films increased again to *E_g_* = 1.44–1.48 eV (Figure 6d). This is similar to the mixed phase of Cu-In-Se materials *E_g_* = 1.17–1.24 eV and to the band gap value of In_2_Se_3_ *E_g_* = 1.55 eV (Table 2). The annealing process allowed selenium to crystallize; therefore, the increase in band gap value could be due to the higher band gap value of selenium.

## 4. Conclusions

The SILAR method was successfully used to deposit Cu_x_Se and In-incorporated Cu_x_Se films. The aqueous solutions of H_2_Se_n_S_2_O_6_-type acids were suitable as a selenium precursor. The films were formed using three steps: the deposition of selenium film, the formation of copper selenide film, and the formation of indium–copper selenide film by incorporating and annealing. A number of phases were identified by XRD analysis: hexagonal klockmannite, cubic indium selenide, hexagonal selenium, indium selenide, and cubic copper indium selenide. A mixture of various compounds in the films was confirmed by XPS analysis. The SEM analysis showed that the films have a dendritic structure, agglomerated and not-well-defined grains, and a film thickness of ~90 μm. The AAS and EDX analyses showed that the films contained similar amounts of selenium and indium, while samples treated at a higher temperature and for a longer duration with the solution of copper(II/I) ions had more copper. The analysis of optical properties showed that the band gap values of copper selenide are 1.28–1.30 eV. The band gap values of indium-incorporated copper selenide increased by 5% (1.32–1.38 eV), and increased again by another 5% after annealing (1.42–1.48 eV). The optical properties of the formed films match the optical properties of copper selenide and indium selenide semiconductors.

## Figures and Tables

**Table 1:** Amount of Cu, Se, and In in In- incorporated Cu_x_Se films on glass.

Copper(II/I) Ion Solution Treatment Conditions		Amount of Elements, μmol/cm^2^		
Temperature, °C	Duration, min	Cu	Se	In
40 °C	10	0.50	2.86	0.26
	20	0.59	2.85	0.27
60 °C	5	0.69	2.95	0.29
	10	0.78	2.89	0.32

**Table 2:** The band gap values of selenium, copper selenide, copper indium selenide, and indium selenide found in the literature.

Semiconductor Material	Band Gap Eg, eV	Reference
Se	1.60 eV	[35]
CuSe	1.80–2.10 eV	[36]
Cu_2-*x*_Se	1.4–2.2 eV	[37]
Cu_2_Se	1.1–1.27 eV	[38]
CuInSe_2_ CuIn_3_Se_5_ CuIn_5_Se_8_	1.04 eV 1.17 eV 1.22–1.24 eV	[39]
In_2_Se_3_	1.55 eV	[40]

## Data Availability

Not applicable.
